# Correction to ‘Genome manipulation by guide-directed Argonaute cleavage’

**DOI:** 10.1093/nar/gkae1150

**Published:** 2024-11-18

This is a correction to: Shan Huang, Kaihang Wang, Stephen L Mayo, Genome manipulation by guide-directed Argonaute cleavage, *Nucleic Acids Research*, Volume 51, Issue 8, 8 May 2023, Pages 4078–4085, https://doi.org/10.1093/nar/gkad188

In the originally published version of this manuscript, a grant number was inadvertently omitted from the Funding section. This should have read: ‘Caltech Rosen Bioengineering Center Award; Shurl and Kay Curci Foundation Award; NIH DP2 Award GM140937. Funding for open access charge: Caltech Rosen Bioengineering Center Award.’

These details have been corrected only in this correction notice to preserve the published version of record.

